# The role of indigenous knowledge in drought risk reduction: A case of communal farmers in South Africa

**DOI:** 10.4102/jamba.v9i1.420

**Published:** 2017-07-27

**Authors:** Fummi Muyambo, Yonas T. Bahta, Andries J. Jordaan

**Affiliations:** 1 Disaster Management Training and Education Centre for Africa, University of the Free State, South Africa; 2 Department of Agricultural Economics, University of the Free State, South Africa

## Abstract

Even though the significance of indigenous knowledge in agriculture is internationally recognised, the role thereof in disaster risk reduction in South Africa is not well documented. This article determined the influence of indigenous knowledge in drought risk reduction in O.R. Tambo district of the Eastern Cape province (South Africa). Primary data were collected from 87 communal farmers through purposive sampling using a structured questionnaire. Focus group discussions were also held with the target group (farmers and extension officers) to gain more information and clarification on indigenous knowledge. The finding indicated that the majority of respondents (64.4%) relied on indigenous knowledge in their farming practice and drought risk reduction. Two-thirds (66.7%) of the respondents revealed that indigenous knowledge contributed to the resilience of farmers towards drought. The respondents unanimously agreed that indigenous knowledge is losing its significance in farming and drought risk reduction, because the younger generation did not value it anymore. Lack of documentation and deterioration of its application by the younger generation were found to be the main challenge for most respondents. The study concluded that indigenous knowledge was still an integral part of agricultural practices, applied drought risk reduction strategies and contributed to resilience against disasters. Based on the findings, the study recommends that indigenous knowledge be compiled, documented and published so that all farmers can learn of effective farming practices, passed on from generation to generation. Community holders of such information are encouraged to make younger generations aware of the benefits of indigenous knowledge to promote its usage.

## Introduction

The independence of South Africa from apartheid in 1994 ushered in a new era where indigenous farming would no longer be alienated by the majority of South Africans (Magoro 2004). Indigenous knowledge is a wisdom that develops within a particular culture and specific geographical area and has been orally transmitted from one generation to another through art, songs, stories and laws (Rusiro, Tshuma & Basikiti 2013). Boven and Morohashi (2002) indicate that indigenous knowledge originates among and is maintained by local people, usually in the rural areas, through their extended interaction with their environment. The use of indigenous knowledge is especially relevant to poor and rural communities that have high illiteracy levels and are unable to access information. Indigenous agricultural knowledge provides a means of dealing with challenging situations. Agricultural decisions made on the basis of indigenous knowledge help farmers prepare and cope with catastrophes (Warren 1991).

The growing recognition of indigenous knowledge in cost-effective and sustainable development by African governments and international development agencies shows the need to explore its significance in drought risk reduction. For example, indigenous knowledge related to agriculture and the environment was internationally recognised following the United Nations Conference on Environment and Development, which was held in Rio de Janeiro in June 1992 (United Nations Environment Programme [UNEP] 2008).

In 2015–2016, most of the southern African region, including the Eastern Cape province of South Africa, was affected by agricultural drought, which caused devastating impacts (Daniels 2016). Small-scale farmers and rural-based communal farmers were especially vulnerable. With insufficient government funding to mitigate the impacts of agricultural drought,^1^ the use of indigenous knowledge systems remains a viable option for developing economies to reduce drought impacts (AgriSA 2016; Iloka 2016).

Mavhura et al. (2013) explored people’s indigenous survival strategies and variations in their ability to cope with floods in Zimbabwe and found that indigenous knowledge played a significant role in reducing the impact of floods. Mercer et al. (2007, 2009) highlighted the need for a specific framework identifying how indigenous and western knowledge may be combined to mitigate the intrinsic effects of environmental processes and therefore reduce the vulnerability of rural indigenous communities to environmental hazards and disasters. Notsi (2012) revealed that an indigenous African farming method protected harvested crops against pests for up to 3 years by using wood ash. They also used creeper crops to control weeds and keep moisture in the soil. Dube and Musi (2002) found that in Swaziland maize used to be stored in an underground pit and would consequently last for more than a season. Similarly, Zimbabwean rural farmers store sweet potatoes in underground pits to preserve the food as well as for safekeeping from thieves (Mutandwa & Gadzirayi 2007). The UNEP (2008) gives examples of the application of indigenous knowledge for agricultural purposes, which include mixed cropping, the use of animal manure to improve soil fertility and the ronjo system practiced by Maasai pastoralists in Tanzania. The ronjo system is a traditional method of dividing the village into pasture zones to conserve pasturelands and prevent drought-borne disasters, thus optimising the available land and conserving moisture and fertility of the soil. None of the authors assess the role of indigenous knowledge with respect to the behaviour of different species (such as when locusts, snakes, cobra, butterflies and bees move in the same direction, when frogs make noise and horses playfully jumping) as indicators of imminent drought. As a result, the current knowledge is insufficient. Therefore, the present study attempts to fill this gap in knowledge and literature.

To the best of our knowledge, studies in South Africa on indigenous knowledge are rare and not well established. In the KwaZulu-Natal province of South Africa, local farmers generated their own sorghum seeds by covering the tender seed heads of selected stalks with grass until harvest time to protect from birds (Boylan 2007). In South Africa, some farmers use sun-dried narrow strips of meat (biltong) as a preservation method. The main objective of the study was to explore the use of indigenous knowledge in agricultural practices and drought risk reduction in O.R. Tambo district in Eastern Cape province of South Africa. The indigenous knowledge explored in this study included the behaviour of different species (such as when locusts, snakes, cobra, butterflies and bees move in the same direction, when frogs make noise and horses playfully jumping) as indicators of imminent drought. Moreover, traditional storage practices (such as big and healthy-looking maize cobs kept for seed and stored) and traditional rituals (rain-making rituals) were explored. This study makes a significant contribution to the gap in literature on indigenous knowledge and drought risk reduction in the southern African region and Africa. The similarity of culture and socio-economic characteristics is considered. The research reported in this article is part of a more comprehensive research project on ‘Vulnerability, adaptation and coping with drought: The case of the commercial and subsistence extensive livestock sector in the Eastern Cape’ (Water Research Commission 2014).

## Study area

**FIGURE 1:**
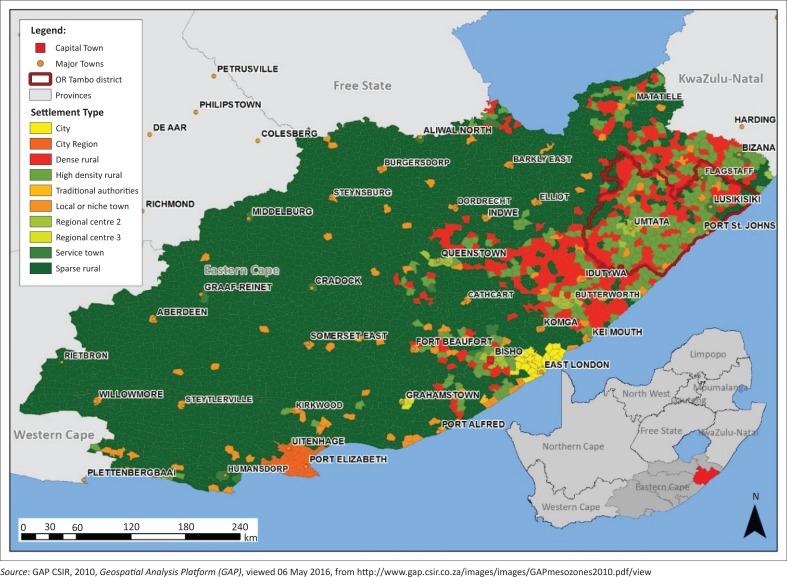
O.R. Tambo district in Eastern Cape province.

O.R. Tambo district is currently one of the most drought-affected districts in the Eastern Cape province of South Africa. The OR Tambo district lies along the eastern side of the Eastern Cape province of South Africa. It stretches along the Indian Ocean coastline for about 160 km and has as its neighbours, KwaZulu-Natal province to the northeast, Joe Gqabi district of Eastern Cape to the northwest, Alfred Nzo district of Eastern Cape to the north, Amathole district of Eastern Cape to the southwest and Chris Hani district of Eastern Cape to the west (Gumenge 2010; Muyambo, Jordaan & Bahta 2017). Figure 1 shows a map of O.R. Tambo district within the Eastern Cape province of South Africa.

Two-thirds (1, 213, 064) of the O.R. Tambo population live in the rural areas, which is rich in Xhosa culture and a high practice of indigenous knowledge. The area is characterised by low income and education levels, under-developed infrastructure, poor access to health, undependable water accessibility, as well as a high level of poverty and unemployment. As a result, communal farmers rely on indigenous knowledge systems (Fobosi 2013; Human Sciences Research Council Report [HSRC] 2012).

## Methodology

To analyse the role of indigenous knowledge systems on drought risk reduction in O.R. Tambo district, we used primary data collected during the period from August to September 2014. Face-to-face interviews and focus group discussions (FCDs) were conducted with 87 communal farmers using a questionnaire to identify the indigenous knowledge indicators used in drought risk reduction in the study area including socio-economic characteristics of respondents; farming practice; types of indigenous knowledge applied such as indicators of early warning signs, preservation techniques for seed, drought preparedness and rituals, scarce animals and challenges faces during application of indigenous knowledge. Homogenous purposive sampling was used to sample communal farmers who shared similar characteristics in terms of their type of farming, as well as their rural background. Workshops were conducted as a platform to administer questionnaires and hold FCDs with the target group. FCDs helped to give more information and clarification on indigenous knowledge indicators. After the workshops, we conducted the face-to-face interviews with communal farmers’ at their homesteads in collaboration with extension officers in the district. The sample for this study was, to a greater extent, influenced by the number of communal farmers who attended the workshops. A total of 89 communal farmers attended the workshops. Out of the 89 farmers, 87 completed the questionnaires. The communal farmers (87) were those who were willing to participate in the survey, after the purpose of the study was explained to them in a workshop. The communal farmers shared similar characteristics in terms of their type of farming, understanding and practice of indigenous knowledge, farm size and communal land ownership. The O.R. Tambo district was selected because people farm on communal land and indigenous knowledge is commonly practiced in the district. The target population comprised all crop and livestock communal farmers in the O.R. Tambo district. As part of gaining an understanding of indigenous knowledge systems on drought risk reduction in the study area, the Rapid Rural Appraisal (RRA) approach was also applied. The RRA is a method of cross-checking information and is a way of obtaining evidential data, especially in an agricultural environment, and was chosen for its flexibility, which made it possible to change focus whenever new information was realised. This method was performed to verify the general information gathered during the interview. The RRA also deals with direct observations of farmers’ lifestyles, their living conditions and any other factors that could influence the role of indigenous knowledge in drought risk reduction.

The survey data collected from the respondents were processed and used to analyse the role of indigenous knowledge. Prior to the analysis, summary statistics of the communal farmers were described to give an overview of the socio-economic characteristics of the respondents. Among the socio-economic characteristics were age, gender, marital status, educational status and farming experience.

## Results and discussion

### Socio-economic characteristics of respondents

Table 1 indicates the socio-economic characteristics of the respondents. The respondents’ median age was 52 years, which, together with the average age of 51 years, shows that the younger generation was not involved in agricultural activities. The average farming experience was 13 years. Although experience is gained with age, farming need not be dominated by aged populations in any region, as this could have negative implications on future food production and adoption of indigenous knowledge (Carino 2013).

**TABLE 1:** Socio-economic characteristic of the respondents.

Variable	Mean	Median	Minimum	Maximum	*n*	%
Age	50.91	52	21	85	-	-
Experience	13.43	10	0.25	60	-	-
**Gender**						
Male	-	-	-	-	62	71
Female	-	-	-	-	25	29
**Marriage status**						
Married	-	-	-	-	64	74
Single	-	-	-	-	14	16
Divorced	-	-	-	-	2	2
Widows	-	-	-	-	7	8
**Schooling/education**						
Not attend	-	-	-	-	23	26
High school	-	-	-	-	44	51
Diploma	-	-	-	-	18	21
First degree	-	-	-	-	2	2

The UNEP (2008) states that it is mostly the older generation who know and use indigenous knowledge to reduce the impact of disasters. The older communal farmers in the study area indicated that they were dependent on indigenous knowledge because it substitutes their limited agricultural resources. They reported difficulty in accessing agricultural resources owing to physical ailments, lack of financial resources which hindered their farm activity, as well as reduced mobility. This corroborates findings by Upreti and Upreti (n.d.) where resource-poor farmers practice indigenous knowledge technologies and rely more on local resources for food production.

This study revealed that more males (71%) were involved in farming than females (29%). The greater proportion of the respondents (74%) were married, whereas 16% were single and 2% were divorced. Widows (8%) comprised a very vulnerable group and may be more susceptible to drought impacts (Cutter et al. 2009). Among the respondents, 26% did not have any schooling at all, of which 40% were women and 21% men. This is higher than the district’s 17.3% illiteracy rate (South Africa, Statistics [StatsSA] 2012). Approximately half (51%) of the respondents attend high school, 21% had a diploma and 2% of the respondents had a first degree. Education is very important in developing a community that is resilient to drought impacts. The lack of education is associated with marginalisation and poverty. The less-educated farmers are, the more they are likely to be susceptible to drought impacts and more likely to apply indigenous knowledge (Adger et al. 2004). Notsi (2012) echoes that indigenous knowledge is important, especially in a rural context such as the study area, with high poverty levels, high unemployment and limited formal education levels. In such circumstances, indigenous knowledge is invaluable, because it is easily accessible without having to invest money to obtain it.

### Indigenous knowledge and drought risk reduction

The results indicated that the majority of respondents (64.4%) relied on indigenous knowledge in their farming practice and drought risk reduction. This finding is in line with studies of Olatokun and Ayanbonde (2008) who found that 44% of respondents used indigenous knowledge in farming and drought risk reduction. Moreover, these findings corroborate those of the UNEP (2008) where the use of indigenous knowledge is still an integral part of most African local communities and agriculture.

Two-thirds (66.7%) of the respondents also revealed that indigenous knowledge contributed to the resilience farmers towards drought. One respondent indicated that indigenous knowledge ‘is wealth in the hands of a Xhosa farmer’. Another respondent stated that ‘indigenous knowledge equips us to prepare for a good harvest or for drought’. Notsi (2012) highlighted that indigenous knowledge is very significant for farmers with limited formal education. The use of indigenous knowledge was relevant to the O.R. Tambo rural community, especially for farmers who were illiterate and could not access information (26% had no schooling). Therefore, indigenous agricultural knowledge helps them to cope with agricultural drought.

### Types of indigenous knowledge applied to drought risk reduction

This section presents some of the indigenous knowledge that communal farmers in O.R. Tambo district in Eastern Cape province of South Africa used in drought risk reduction.

#### Drought early warning signs

A community that lacks drought-related information and early warning systems, whether traditional knowledge or access to media and other communication systems, is more vulnerable to drought impacts (Wongbusarakum & Loper 2011). A disaster risk reduction plan is critical in determining the resiliency of a community to hazards, as is having early warning systems for the community, which increases the capacity to reduce drought impacts (Asian Disaster Prevention Centre 2006). Through interview and FCDs, O.R. Tambo district communal farmers revealed that during the year, when they saw an army of locusts moving in the same direction, they interpreted or knew that drought was imminent. Farmers explained that the forthcoming drought and rain could be predicted from the behaviour of different species as shown in Table 2.

**TABLE 2:** Behaviour of different species as a sign of drought and rain.

Species	Behaviour	Description in relation to drought or rain
Snakes	If they see snakes moving in the same direction	It will signify drought
Bees	When bees fly in a certain direction	It will signify drought
Frogs	When frogs make much noise in the afternoon	It will signify drought
Horse	When a horse playfully jumps	It will signify rain
Butterflies	When a kaleidoscope of butterflies fly together	It will signify drought a good farming season

#### Preservation of seed and production

Most of the respondents (80%) from FCDs indicated that the elderly people check the maize while still in the field for good quality cobs. The big maize cobs are guarded strictly and reserved for seed. During harvesting, the big maize cobs are set aside and kept for seed for the following growing season. Some of the maize cobs are kept in their round huts, locally known as *Intanyongo* or *Iziswenye*. The maize cobs are hung from the ceiling to ensure complete dryness. Some farmers sprinkle some ash around the seed to keep ants away.

They further explained that if the production of maize and sorghum was good enough, farmers would take some of the bags after harvesting and keep them in big water tanks as a reserve for difficult periods. They added that their grandfathers used to dig a big deep hole in the middle of the *kraal* (homestead) as a means of storing excess food or production. This would prevent thieves from gaining access to the food. They could retrieve the food with care when needed, thus ensuring food security.

#### Drought preparedness and rituals

The respondents from FCDs indicated that the presence of ants all over the place during dry periods signified that worse times are still to come, and the leaders of villages used to announce at the chief’s meeting. The respondents indicated that the village leaders would set a date and time to go up a sacred mountain such as *Qwempe*. They would conduct traditional dances called *Imingqungqo* or *umxhentso wakwantu* in IsiXhosa (one of the languages in Eastern Cape province of South Africa). The people would carry along traditional beer and would slaughter one of their cattle. In doing so, a request was passed to their ancestors. Some would use a big river to perform a similar ritual. *Ngonyama* river in Eastern Cape province of South Africa is one such sacred river. Usually, after that ceremony, the rains would come. The respondent who explained this ritual added by stating that ‘the present generation does not believe nor bother themselves about these important rituals which used to help reduce incidents of drought’.

The practice of conducting ceremonies for rain is no longer as common in the villages as it used to be a few years ago. Sacred ceremonies were also practiced in other African countries like Zimbabwe (*Inyangani* mountain) (Muleya 2014; News Day 2014; Ngara & Mangizvo 2013), Lesotho and in the Eastern Cape province of South Africa. There are sacred hills, forests and wells where they performed ceremonies to their ancestors before and after harvest and ancestral spirits would give rain in answer to their prayers (Rusiro, Tshuma & Basikiti 2013).

#### Sacred animals

According to the respondents in this study, the Eastern Cape province of South Africa believes that a brown animal, such as a brown swiss (cattle breed, which is good for beef and milk production), should not be kept among their cattle, because they bring bad luck (affecting breeding) to the herd of cattle. On another note, two respondents concurred that ‘some people believe that the skin of a rabbit should not be put in the fire, because they believed it would cause dryness; rains would fail which may lead to drought’. However, the accuracy of these predictions (brown animal and skin of rabbit) seem mythical. Some animals and birds are sacred across African indigenous communities such as a lone baboon, which is an ancestral symbol among the *Shangwe* people in Gokwe, Zimbabwe. There are also lions that do not attack local people and are associated with rain messages (Ngara & Mangizvo 2013).

#### Challenges in using indigenous knowledge

The respondents unanimously agreed that indigenous knowledge is losing its significance in farming and drought risk reduction, because the younger generation does not value it anymore. Places, animals and practices that used to be sacred and taboo are no longer regarded in that manner. In agreement with Boven and Morohashi (2002), poor documentation of indigenous knowledge was mentioned as one of the contributing factors to the decline of indigenous knowledge use. The respondents further blamed the advent of western science as a major cause for the deterioration of indigenous knowledge.

This finding is in line with Rusiro, Tshuma and Basikiti (2013) who argued that western science is considered to be more superior and civilised than traditional knowledge. On the contrary, traditional knowledge is considered demonic, inferior and mythical. They claimed that the younger and more formally educated generation are embarrassed to be associated with it. Similar challenges have been highlighted by other scholars such as Dube and Musi (2002) and Daniels (2016) who found that people, particularly the youth, have a negative attitude towards indigenous knowledge.

## Conclusion

The majority of O.R. Tambo district population was found to be living in the rural areas where indigenous knowledge is deemed to be important because of general lack of resources. Most of the respondents relied on indigenous knowledge in their farming practice. They considered indigenous knowledge as an integral part of agricultural practices that was applied in drought risk reduction and contributed to communal farmer’s resilience against disasters.

The study also looked at the challenges in effective use of indigenous knowledge, which included poor documentation and deterioration of its application by the younger generation. Because of the value and contribution of indigenous knowledge in drought risk reduction and resilience against disasters, this study recommends that indigenous knowledge in the O.R. Tambo district be compiled, documented and published. This will give access to the knowledge to some farmers who are not aware of it so that they may learn of effective indigenous farming practices, passed on from generation to generation. Community holders of indigenous knowledge are encouraged to make the younger generation aware of the knowledge in order to promote its usage and continued passage of it from generation to generation.

## Footnotes

1 *Agricultural drought* is a shortage of precipitation during the growing season impinges on crop production or ecosystem function in general because of soil moisture drought (IPCC 2012).
